# 

**Published:** 1899-04-08

**Authors:** 


**Notices of Births, Deaths, and Marriages** are inserted in this column at a charge of 2s. 6d. for an announcement not exceeding four lines.

### NOTICE TO CORRESPONDENTS.

All MS., Letters, Books for Review, and other matters intended for the Editor should be addressed The Editor, "The Hostital," The Hospital Building, 28 & 29, Southampton Street, Strand, W.C.

The Editor cannot undertake to return rejected MS., even when accompanied by stamped directed envelope.

**Notice to Advertisers and Subscribers.**

All Advertisements, Orders for Copies of The Hospital, and Business Communications should be addressed to **THE MANAGER (Not to the Editor)**, The Hospital, The Hospital Building, 28 & 29, Southampton Street, Strand, London, W.C.

The Cost of Subscription, post free, is as follows:—Per week, 2½d.; per quarter, 2s. 8d.; per half-year, 5s. 3d.; per annum, 10s. 6d. Foreign:—Per annum, 15s. 2d., payable, in all cases, in advance.

**NOTICE.—Vol. XXV. of "The Hospital" (October, 1898, to March, 1899), in handsome cloth binding, will shortly be on sale at the Office of this Journal, price 7s. 6d. Cases for binding the Numbers contained in this Volume 1s. 6d. Index to Vol. XXV., 2½d., post free.**

**The Monthly Part for April is now ready. Price 6d., by post 8d.**

### BOOKS RECEIVED.

Rivingtons.

"Remedies for the Needless Injury to Children Involved in the Present System of School Education." By Clement Dukes, M.D. Lond.

Smith, Elder, and Co.

"Growing Children: Their Clothes and Deformity." By Noble Smith, F.R.C.S. Edin.

Open Court Publishing Company and Kegan Paul, Trench, Trubner, and Co.

"The Principles of Bacteriology." By Dr. Ferdinand Hueppe; translated by Dr. E. O. Jordan.

The Sanitary Publishing Company.

"The Cry of the Children; or, The Anti-Vaccinationists." By Mrs. Bray.

E. Sproston, Manchester.

"Biblical Midwifery." By R. M. Fenn, M.B., C.M.

Albert E. Evans.

"Descriptive Catalogue of Orthopædic Apparatus."

J. A. Preuss, Zurich.

"Ragatz Pfäfers."

Sampson Low, Marston, and Co.

"Twentieth Century Practice." Edited by Thomas L. Stedman, M.D. Vol. XVII.

J. B. Lippincott and Co.

"An Experimental Research into Surgical Shock." By George W. Crile, A.M., M.D., Ph.D.

Neill and Co., Edinburgh.

"Saline Irrigations in Abdominal Operations." By George A. Hawkins-Ambler, F.R.C.S.E.

